# Diverse viral glycoproteins as well as CD4 co-package into the same human immunodeficiency virus (HIV-1) particles

**DOI:** 10.1186/1742-4690-11-28

**Published:** 2014-04-03

**Authors:** Devon A Gregory, Grace Y Olinger, Tiffany M Lucas, Marc C Johnson

**Affiliations:** 1 Department of Molecular Microbiology and Immunology, University of Missouri, Columbia, MO, USA; 2 Boston University School of Medicine, Boston, MA, USA; 3 Washington University School of Medicine, St. Louis, MO, USA

**Keywords:** Env, Assembly, Pseudotype, Glycoprotein recruitment, CD4, VSV-G, Ebola GP

## Abstract

**Background:**

Retroviruses can acquire not only their own glycoproteins as they bud from the cellular membrane, but also some cellular and foreign viral glycoproteins. Many of these non-native glycoproteins are actively recruited to budding virions, particularly other viral glycoproteins. This observation suggests that there may be a conserved mechanism underlying the recruitment of glycoproteins into viruses. If a conserved mechanism is used, diverse glycoproteins should localize to a single budding retroviral particle. On the other hand, if viral glycoproteins have divergent mechanisms for recruitment, the different glycoproteins could segregate into different particles.

**Results:**

To determine if co-packaging occurs among different glycoproteins, we designed an assay that combines virion antibody capture and a determination of infectivity based on a luciferase reporter. Virions were bound to a plate with an antibody against one glycoprotein, and then the infectivity was measured with cells that allow entry only with a second glycoprotein. We tested pairings of glycoproteins from HIV, murine leukemia virus (MLV), Rous sarcoma virus (RSV), vesicular stomatitis virus (VSV), and Ebola virus. The results showed that glycoproteins that were actively recruited into virions were co-packaged efficiently with each other. We also tested cellular proteins and found CD4 also had a similar correlation between active recruitment and efficient co-packaging, but other cellular proteins did not.

**Conclusion:**

Glycoproteins that are actively incorporated into HIV-1 virions are efficiently co-packaged into the same virus particles, suggesting that the same general mechanism for recruitment may act in many viruses.

## Background

HIV-1, like other enveloped viruses, can incorporate glycoproteins from different viruses during assembly, allowing for an alternative tropism of the formed virion, a phenomenon known as pseudotyping [1-3]. Despite pseudotyping being a useful tool for gene delivery in both research and therapy, the mechanism of pseudotyping is poorly understood. In some instances, incorporation may be coincidental, as any glycoprotein present in the membrane that a virion collects during assembly might be incorporated in a passive manner. However, with some retrovirus and glycoprotein pseudotype combinations, incorporation appears to be an active process [4]. This observation raises questions about whether many enveloped viruses share a basic conserved mechanism for acquiring viral glycoproteins during assembly and what such a mechanism might be.

Incorporation of HIV Env into HIV virions is generally thought to occur due to physical interactions between the cytoplasmic tail domain (CTD) of the glycoprotein and the matrix (MA) domain of Gag (reviewed in [5,6]). However, for pseudotyped viruses, such an interaction is unlikely except when the viral proteins are from very closely related viruses that can exchange structural components, such as HIV-1 and HIV-2 [7]. Even for native Env recruitment, physical interactions mediated by the CTD of Env are not always necessary. For example, deletion of MLV Env’s CTD does not prevent its enrichment at viral assembly sites [8]. While the CTD of HIV Env is required for Env incorporation into HIV particles in some cell types, it is not required in others, such as human embryonic kidney (HEK) 293 cells [9,10]. Deletion of HIV-1 Env’s CTD allows it to efficiently pseudotype with virions other than HIV-1 and to be functional with some HIV-1 Gag MA mutations that are incompatible with full length Env [11-14]. Similarly, the HIV-1 MA domain appears to be important only for native Env incorporation. Deleting most of the MA domain or replacing the MA domain with heterologous membrane binding domains still allows efficient pseudotyping of HIV-1 by MLV Env and VSV-G [15-18]. Thus it is unlikely that a direct physical interaction between viral glycoproteins and Gag is the primary mechanism of active pseudotyping.

Physical interactions mediated by another protein comprise another possible mechanism of glycoprotein recruitment. Such an intermediate, tail-interacting protein (TIP) 47, has been proposed for native HIV-1 Env recruitment, though its involvement has been called into question and may act only in specific cell types [19-21]. However, for pseudotyping combinations such as HIV virions and MLV Env, where the domains most likely to be involved in such indirect interaction are dispensable, a simple protein intermediate is also unlikely.

Another candidate for an intermediary of glycoprotein recruitment is the lipid membrane microdomain of a virion. Many viruses have been shown to acquire lipids that are characteristic of lipid rafts [22-24]. Recent studies on the membranes of HIV-1 virions have shown them to have a unique composition that shares features of lipid rafts and tetraspanin enriched microdomains [25-28]. Thus, an attractive model for glycoprotein recruitment is that the unique membrane microdomain(s) of an assembling virion has specific features that attract and/or retain viral glycoproteins. Consistent with this model, Leung et al. reported that HIV-1 Env and Ebola glycoprotein (GP) are present in separate microdomains and are incorporated into separate individual HIV-1 virions [29].

If some viruses share a common mechanism for glycoprotein incorporation, one would expect those viruses to have their glycoproteins efficiently incorporated into the same individual pseudotyped virion. When mechanisms differ, for example if different microdomains are used or if specific direct protein interactions are used, the glycoproteins may not be compatible with the same individual virion, though this will not always be the case. To determine which glycoproteins might share the same mechanism, or at least have compatible mechanisms, for incorporation, we sought to identify glycoprotein pairs that could be efficiently incorporated into the same individual HIV-1 virion. We found a correlation between the ability of a glycoprotein to efficiently pseudotype HIV-1 particles and its ability to co-assemble with a different glycoprotein into the same virion.

## Results & discussion

### Visualization of glycoprotein recruitment by SEM

We have previously shown that the distribution of glycoproteins relative to viral assembly sites can be imaged by scanning electron microscopy (SEM) [4]. MLV Env and VSV-G are two glycoproteins that efficiently form infectious pseudotyped particles with HIV-1 and that also appear highly enriched at HIV-1 assembly sites by SEM. In contrast, RSV Env forms infectious pseudotyped particles less efficiently with HIV-1 and is not seen to be enriched at HIV-1 assembly sites by SEM despite efficient surface expression. By high resolution fluorescence microscopy, HIV-1 Env has been reported to be concentrated on HIV-1 particles [30,31]. We wished to determine if HIV-1 Env can also be visualized by SEM, if it is enriched at viral assembly sites, and if MLV Env is enriched at the same sites. Successful labeling of both glycoproteins on the same virion using different sized gold particles would be an indication that the mechanisms of recruitment of the two glycoproteins are compatible and may be the same.

**Figure 1:**
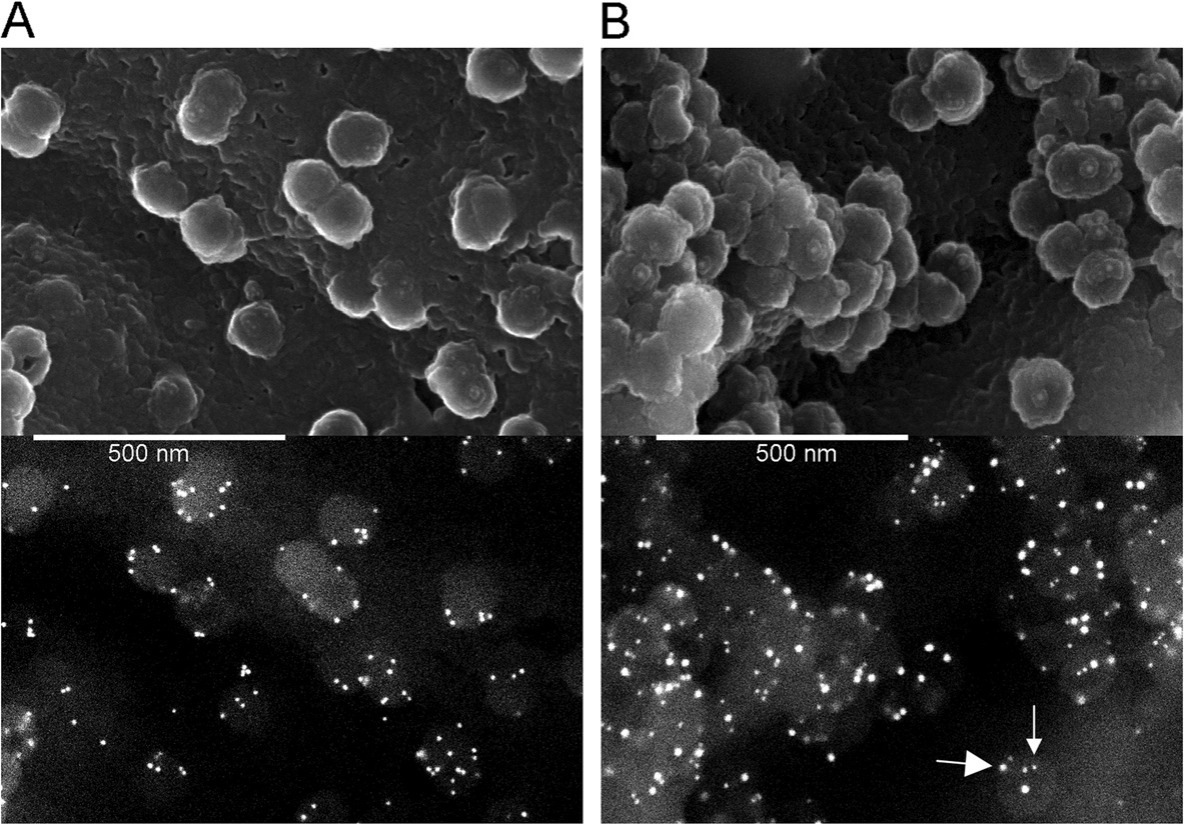
**SEM of HIV Env and MLV Env recruitment to viral particles. A)** Cells producing HIV-1 Gag and GagPol without functional late or PR domains, and producing HIV-1 Env were labeled with 10 nm immunogold against HIV-1 Env and then imaged by SEM. **B)** Cells as in A, but also producing MLV Env were labeled with 10 nm (small arrow) and 18 nm (large arrow) immunogold against HIV-1 and MLV Envs, respectively, and then imaged by SEM.

Cells plated on a glass coverslip were co-transfected with plasmids encoding HIV-1 Env and/or MLV Env, together with a plasmid containing an HIV-1 provirus lacking *Env* and encoding mutated late and protease domains. A day later the cells were prepared as outlined in the Methods and then visualized by SEM (Figure 1). Immunogold labeled HIV-1 Env was seen enriched on budding virions compared with other regions of the plasma membrane (Figure 1A). When both glycoproteins were produced, both were enriched on virus particles with no discernable segregation (Figure 1B). Cross-reaction of the labeling antibodies was not observed (data not shown). This result indicates that the recruitment mechanisms for both HIV and MLV glycoproteins into HIV-1 particles are compatible with each other in this cell type.

### Viral capture and infectivity assay for co-assembly of glycoproteins

**Figure 2:**
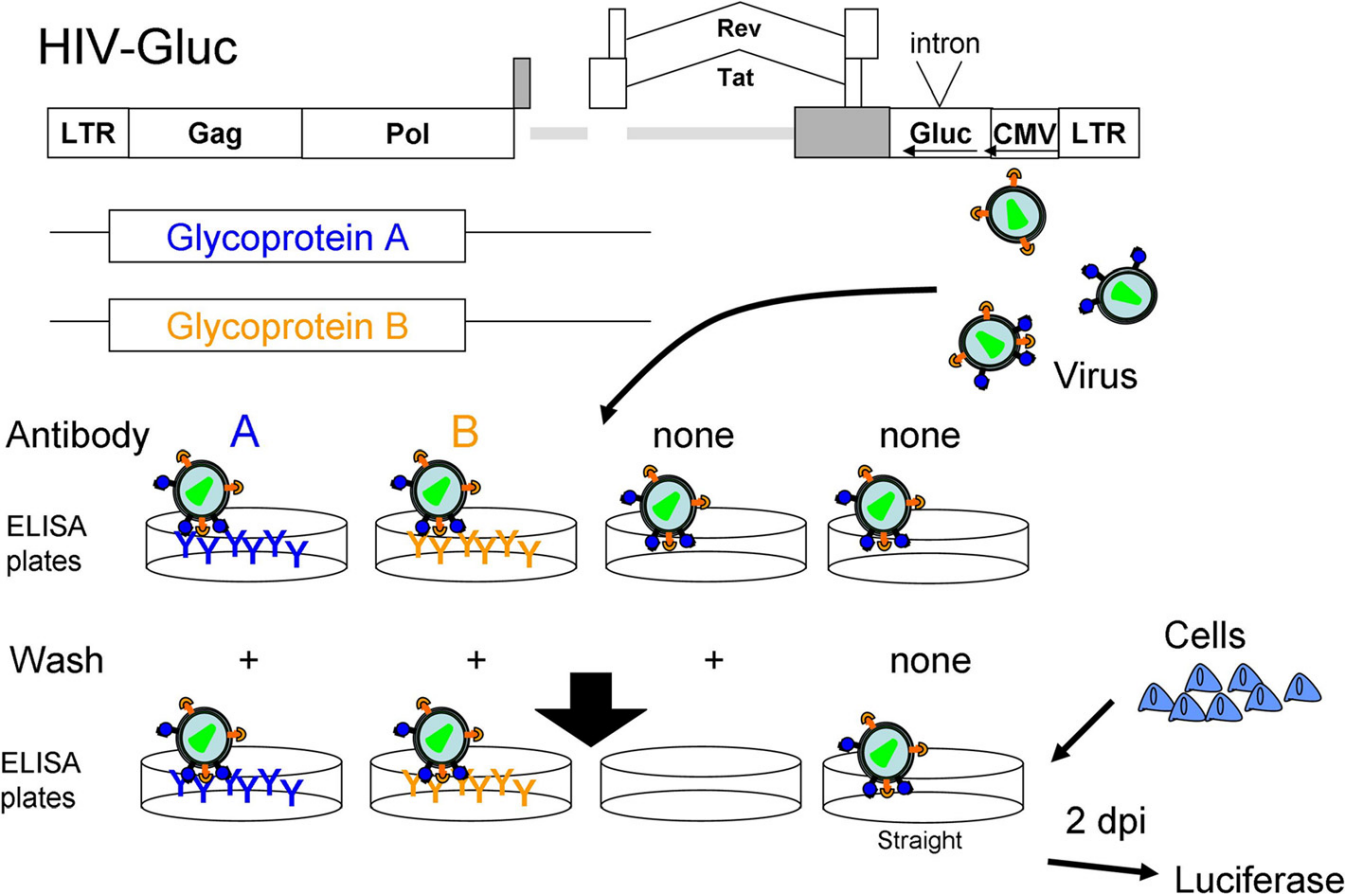
**Schematic of co-capture assay.** The minimal HIV-1 provirus with an intron interrupted reverse *Gaussia* luciferase gene as a reporter and the desired glycoproteins were transfected into 293FT cells to produce virus. The virus was then captured by antibodies against the glycoproteins or no antibody in the wells of an ELISA plate. Unbound virus was washed away from all wells except a straight infectivity positive control. Cells susceptible to infection mediated by one or the other of the glycoproteins were then added to the wells and two days later luciferase production was assayed.

Though SEM can show co-incorporation of different glycoproteins into the same virus particle, it is a technically challenging assay that is inherently qualitative. In order to have a more efficient and quantitative assay for co-packaging of glycoproteins, we developed a variation on the traditional antibody-mediated virus capture assay (Figure 2) [32,33]. To perform this assay, viruses from a provirus with an intron-interrupted, reverse *Gaussia* luciferase gene were produced from cells expressing an individual viral glycoprotein or a pair of viral glycoproteins. The intron-interrupted luciferase gene insured that only cells infected with the virus, and not any transfected cells, would produce active luciferase [31]. The concentration of plasmids for each glycoprotein was selected to ensure that the glycoprotein was the rate-limiting component for infectious particle production and that the glycoproteins all produced roughly equivalent numbers of infectious particles (data not shown). Antibodies to either of the two glycoproteins were bound to wells of an enzyme-linked immunosorbent assay (ELISA) plate followed by addition of the virus. After allowing virus to be captured by the antibodies, free virus was washed away with PBS. Cells permissive to virus with one of the two glycoproteins were then seeded into the wells. After two days, infection efficiency was quantified by detection of luciferase activity. The infection efficiency of virus captured with antibody against the infection incompetent glycoprotein was used to measure the co-packaging efficiency. For controls, viruses were added to wells with no antibody and either washed away (no antibody capture) or not washed away (straight infection) prior to cell seeding. This assay will be referred to as a co-capture assay from here on.

**Figure 3:**
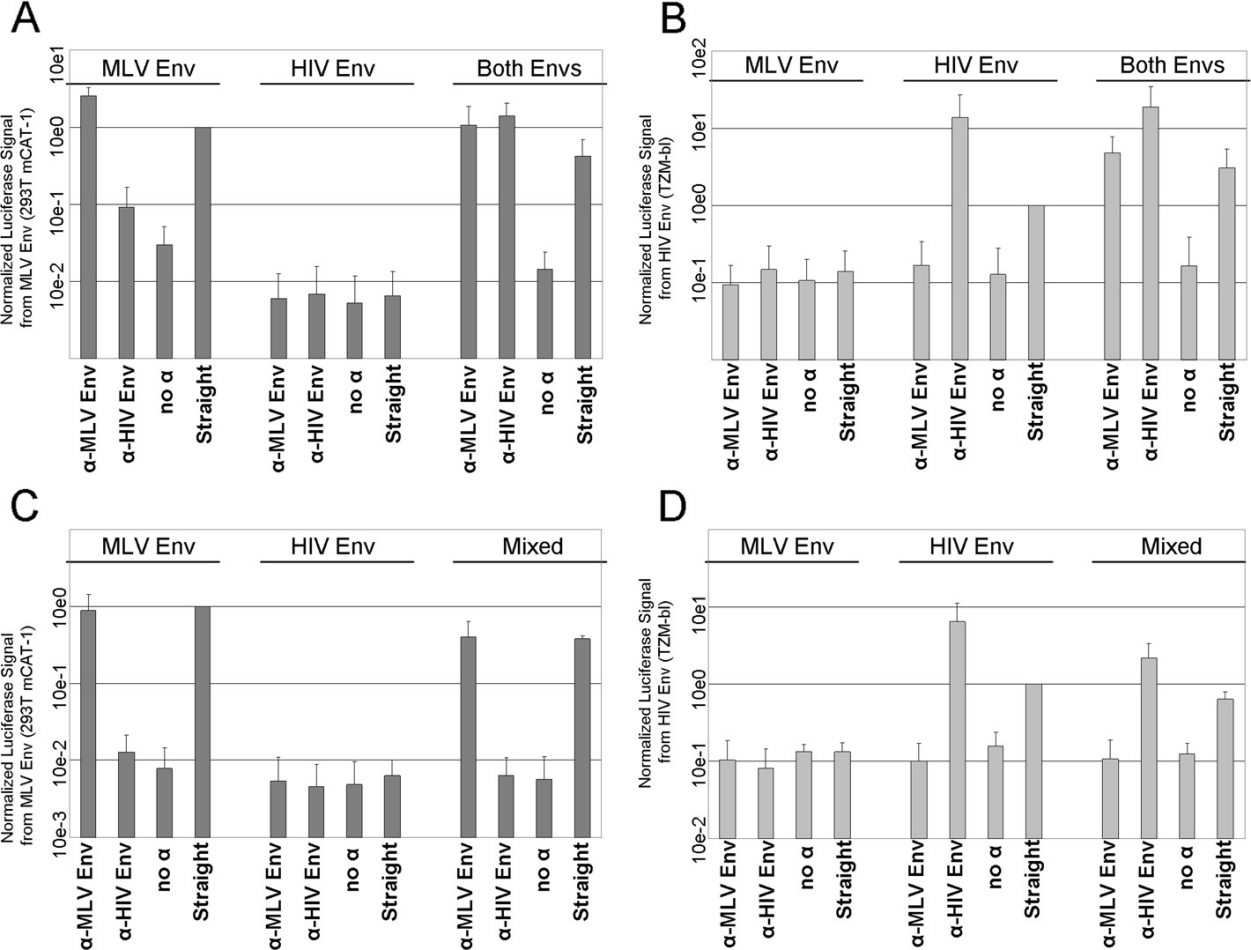
**Co-capture of HIV-1 Env and MLV Env. A & B)** Co-capture was performed using virus with HIV-1 Env and MLV Env. Luciferase signal from **A)** 293 T mCAT-1 cells or **B)** TZM-bl cells infected with virus produced in the presence of MLV Env, HIV-1 Env or both Envs and then subjected to the following: anti-MLV Env capture + wash, anti-HIV capture + wash, no antibody capture + wash & straight infection (no antibody, no wash). The signal was normalized to the straight infection positive control from the relevant single glycoprotein virus sample. All graphs are the average of at least three independent experiments unless indicated otherwise and show standard deviation. **C & D)** Luciferase signal as before from **C)** 293 T mCAT-1 and **D)** TZM-bl cells infected with virus produced with only MLV Env, only HIV Env or a mixture of the two viral supernatants. **A & B** were the average of at least 3 independent experiments, **C & D** were the average of two experiments.

We first tested MLV Env and HIV Env with the co-capture assay to see if the result from SEM could be recapitulated (Figure 3A & B). Using virus produced only with MLV Env, infection of 293 T cells expressing the MLV Env receptor, mouse cationic amino acid transporter protein 1 (mCAT-1), after virus capture with an antibody against MLV Env yielded a luciferase signal 2.5-fold higher than with straight infection, indicating efficient capture (Figure 3A). The greater efficiency from the capture compared with straight infection was frequently observed, and we suspect that it is due to immobilized virus having a greater chance of contact with cells compared with free virus. Virus captured with antibody against HIV Env or without any antibody yielded signal 0.08- and 0.03-times that of straight infection, respectively. Virus produced with HIV Env alone cannot infect 293 T mCAT-1 cells as they lack CD4, the receptor for HIV Env, and thus this virus resulted in no luciferase signal under any of the conditions. Virus produced with both MLV Env and HIV Env was efficiently captured with antibody against either glycoprotein. Capture of this virus with an antibody against MLV Env or HIV Env yielded luciferase signal 2.3- or 3.6-fold higher than straight infection, respectively. Capture with no antibody yielded a signal that was 0.05-times that of straight infection.

In the reciprocal cell line which expresses CD4, TZM-bl, virus produced with HIV Env alone yielded a luciferase signal when captured with antibody against HIV Env, but not when captured with antibody against MLV Env or no antibody (13.8-fold, 0.17-times & 0.13-times that of straight infection, respectively) (Figure 3B). Virus produced with MLV Env alone cannot infect TZM-bl cells as these cells lack mCAT-1, the receptor for MLV Env, and thus this virus yielded in no luciferase signal under any of the conditions. Virus produced with both glycoproteins could be captured with antibody against either HIV Env or MLV Env (6.5- and 1.9-fold of straight). Capture with no antibody yielded a signal that was 0.07-times that of straight infection. These results indicate that HIV Env and MLV Env are present in the same individual virions, consistent with what we observed by SEM.

To rule out the possibility that co-capture infection was occurring due to viral particles with different glycoproteins stuck to each other, and not from glycoproteins in the same individual virion, we mixed supernatants that contained viruses produced with only one or the other glycoprotein and performed the co-capture assay (Figure 3C & D). With the mixed viruses, we did not observe co-capture. Virus captured with antibody against HIV Env did not produce a signal with 293 T mCAT-1 cells, and virus captured with antibody against MLV Env did not produce a signal with TZM-bl cells. Thus it is unlikely that virus was captured indirectly by one virus sticking to another, and thus the signal we observed from virus produced with both Envs was in fact due to both glycoproteins being present in the same virion.

**Figure 4:**
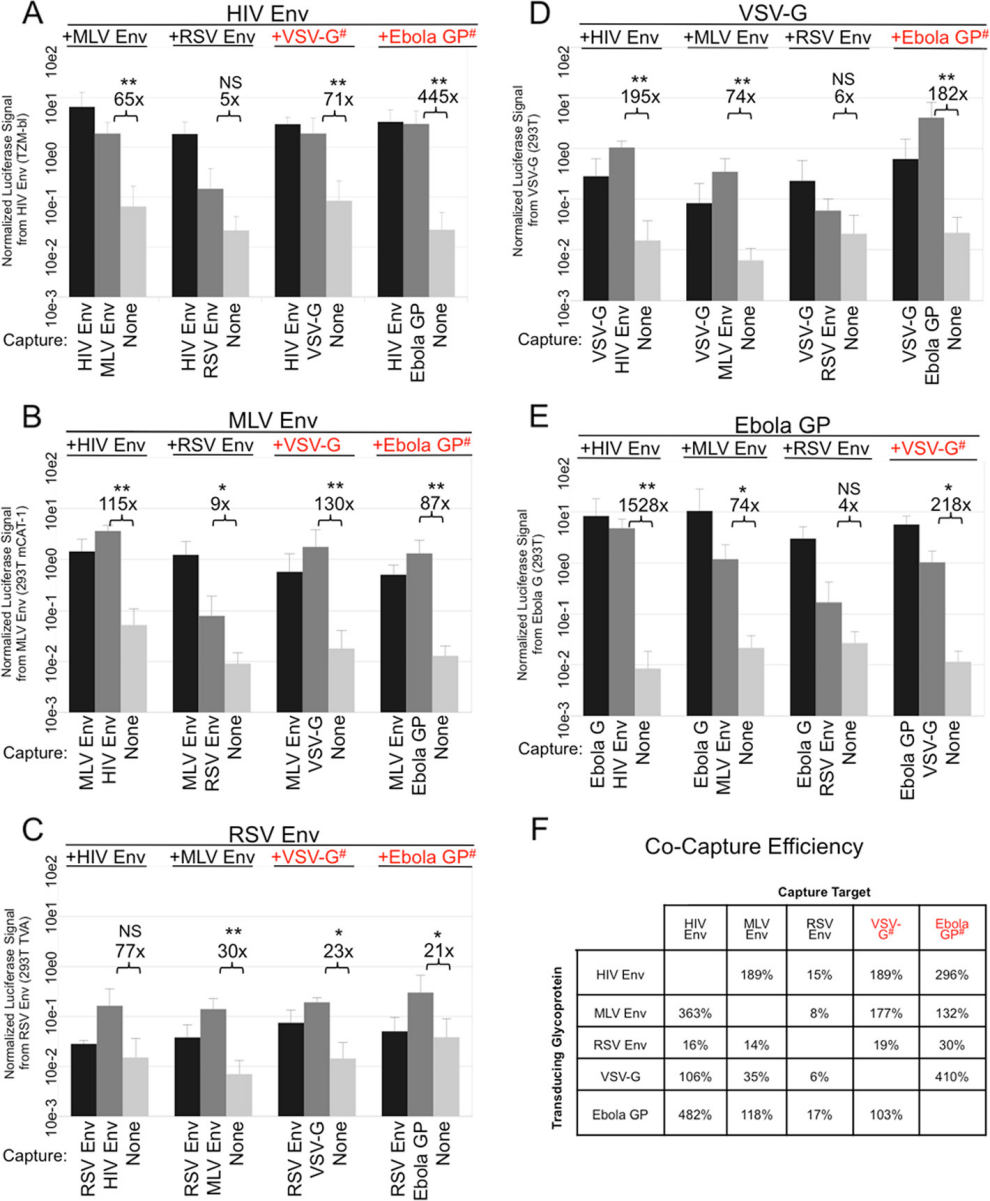
**Diverse viral glycoproteins can be co-packaged.** Co-capture was performed using all paired combinations of HIV-1 Env, MLV Env, RSV Env, VSV-G and Ebola GP. Graphs show the luciferase signal from infection mediated by **A)** HIV-1 Env in TZM-bl cells, **B)** MLV Env in 293 T mCAT-1 cells, **C)** RSV Env in 293 T TVA cells, **D)** VSV-G in 293 T-derived cells or **E)** Ebola GP in 293 T-derived cells when virus was produced with the above glycoproteins and then captured with the indicated antibody. Red text with a # indicate the use of an entry defective VSV-G or Ebola GP as the target for capture. Signal was normalized to the straight infection for each experiment prior to averaging. The average fold increase from antibody capture/no antibody capture is shown for each co-capture sample. **F)** The average percent capture for each paring relative to the straight infection is shown. All graphs are the average of at least three independent experiments and standard deviations are shown. *p < 0.05; **p < 0.01; NS p > 0.05.

We performed this co-capture assay for all pairings of HIV Env, MLV Env, RSV Env, VSV-G and Ebola GP (Figure 4A-F). As before, virus produced with only one glycoprotein was not captured with antibodies against other glycoproteins, and could only infect specific target cell lines (data not shown). The exceptions to the latter are VSV-G and Ebola GP, which mediate infection of a wide range of cell lines, including all the ones we used here. So that these glycoproteins would not mediate infection when targeted for capture, we used entry defective mutants of VSV-G (A117R) and Ebola GP (G87A and F88A) in parallel to the wildtypes [34,35]. All glycoproteins were efficiently co-packaged except for RSV Env. With all pairings of HIV Env, MLV Env, VSV-G and Ebola GP there was significant enrichment of infectivity with the reciprocal antibody compared to the no antibody control (Figure 4A-E).

The antibody used to capture RSV Env-containing viruses appeared to be less efficient at self-capture, which casts doubt on its apparent inefficiency at co-capture (Figure 4C). RSV Env is not actively recruited to HIV-1 virions despite efficient surface expression and thus inefficiently incorporated during assembly, likely making virions very sensitive to neutralization by the capture antibody [4]. However, RSV Env co-capture was also inefficient when virus was captured with antibodies against the reciprocal glycoproteins (Figure 4A, B, D-F). Since the other antibodies were efficient at self-capture, these data indicate that RSV Env is not co-packaged efficiently with the other viral glycoproteins (Figure 4C).

While all the glycoproteins other than RSV Env were efficiently co-packaged with each other, capture of MLV Env and VSV-G-containing viruses with antibody against MLV Env, followed by infection with VSV-G was 0.35-fold that of straight infection (Figure 4D & F), while the reciprocal was 1.77-fold that of straight (Figure 4B & F). Though capture of VSV-G containing virus with antibody against MLV Env was generally inefficiently, it was still 5-fold greater than capture with an antibody against RSV Env. Since MLV Env and VSV-G-containing viruses were efficiently captured with antibody against VSV-G, the data overall suggest MLV Env and VSV-G do efficiently co-package, if slightly less efficiently than other pairings (Figure 4B & F). In total, the data from the co-capture assay suggest that co-packaging is most efficient among glycoproteins that are actively recruited to viral assembly sites.

We did not expect Ebola GP to be co-packaged with HIV Env, as these two glycoproteins have been reported to segregate into distinct particles [29]. However, we found that Ebola GP is co-packaged with all of the glycoproteins tested except for RSV Env, as noted above (Figure 4A-F). The reason for this discrepancy with the previous study is not readily apparent and may simply reflect differences in reagents or assay methods. Ebola GP is known to form pseudotyped virions effectively with HIV-1 suggesting that it is actively recruited into the assembling particles [36]. That Ebola GP is efficiently co-packaged with the glycoproteins that are actively recruited during assembly also suggests that it is actively recruited.

### Cellular membrane proteins co-packaging efficiency

**Figure 5:**
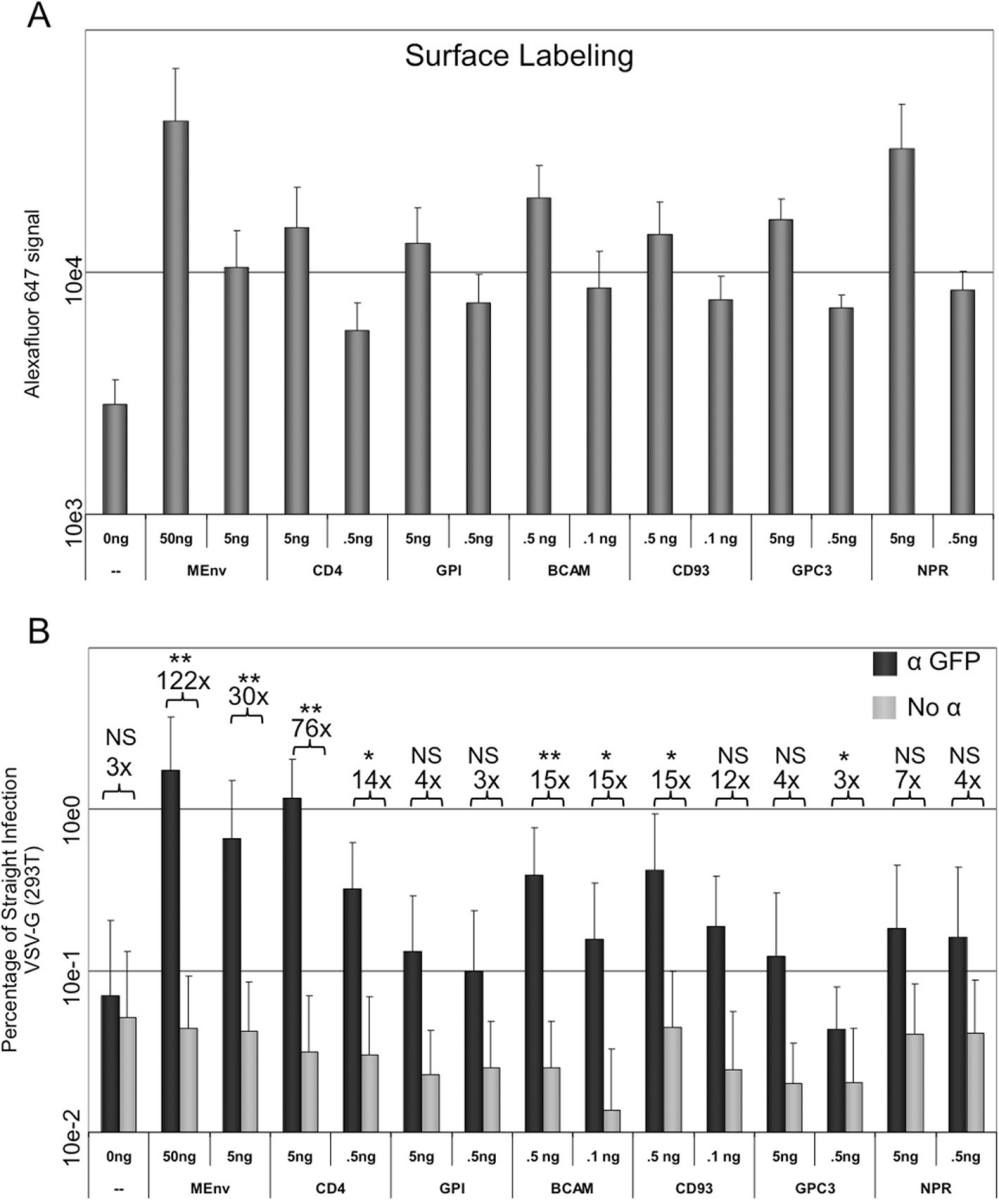
**Co-capture using cellular glycoproteins.** Co-capture was performed on virus produced from cells transfected as before using VSV-G and the indicated amounts of GFP-tagged cellular proteins or YFP-tagged MLV Env. **A)** The surface expression of the indicated proteins in transfected cells was assayed by surface labeling GFP with an anti-GFP antibody conjugated to Alexa Fluor 647, which was detected by flow cytometry. **B)** The luciferase signal from the anti-GFP captured samples and the no-antibody captured samples normalized to the straight control is shown. The average fold increase of antibody/no antibody is shown for each pair. All graphs are the average of at least three independent experiments and standard deviations are shown. *p < 0.05; **p < 0.01; NS p > 0.05.

We wished to determine if cellular proteins were co-packaged in a similar manner as observed with the viral glycoproteins. To test co-packaging of cellular proteins, we constructed expression vectors to produce the GFP-tagged cellular membrane proteins CD4, basal cell adhesion molecule (BCAM), CD93, Glypican-3 (GPC3) and Natriuretic Peptide Receptor C (NPR), as well as a GPI-anchored GFP. This collection of proteins was chosen to include single pass type I transmembrane proteins with short cytoplasmic tails and GPI-anchored proteins. CD4, BCAM, CD93, GPC3 and NPR have predicted cytoplasmic tails 38, 56, 45, 8 and 37 amino acids in length, respectively. GPI anchored proteins are incorporated into HIV-1 particles, but reports have varied on the efficiency of incorporation [25,26,37-42]. CD4 is known to be efficiently incorporated into HIV-1 virions, but it has not been shown to be actively recruited [43,44]. The production and surface localization of each of these proteins, along with the YFP-tagged MLV Env, was verified by fluorescence detection of total GFP/YFP and Alexa Fluor 647 labeled surface GFP/YFP by flow cytometry (Figure 5A). Concentrations for each cellular glycoprotein expression plasmid were adjusted to result in similar surface labeling as 5 ng of the MLV Env plasmid used previously. The co-capture assay was then performed with each of these cellular proteins and VSV-G (Figure 5B). Capture efficiency was greater when more plasmid was used in the transfection for each protein, but differences in efficiency were observed among the different proteins. Of the cellular proteins, CD4 appeared to be co-packaged with VSV-G the most efficiently, followed BCAM, CD93, GPI, NPR and GPC3. Although the mean viral capture was higher than the no antibody control with all of the cellular proteins, in many cases the enhancement was not statistically significant.

**Figure 6:**
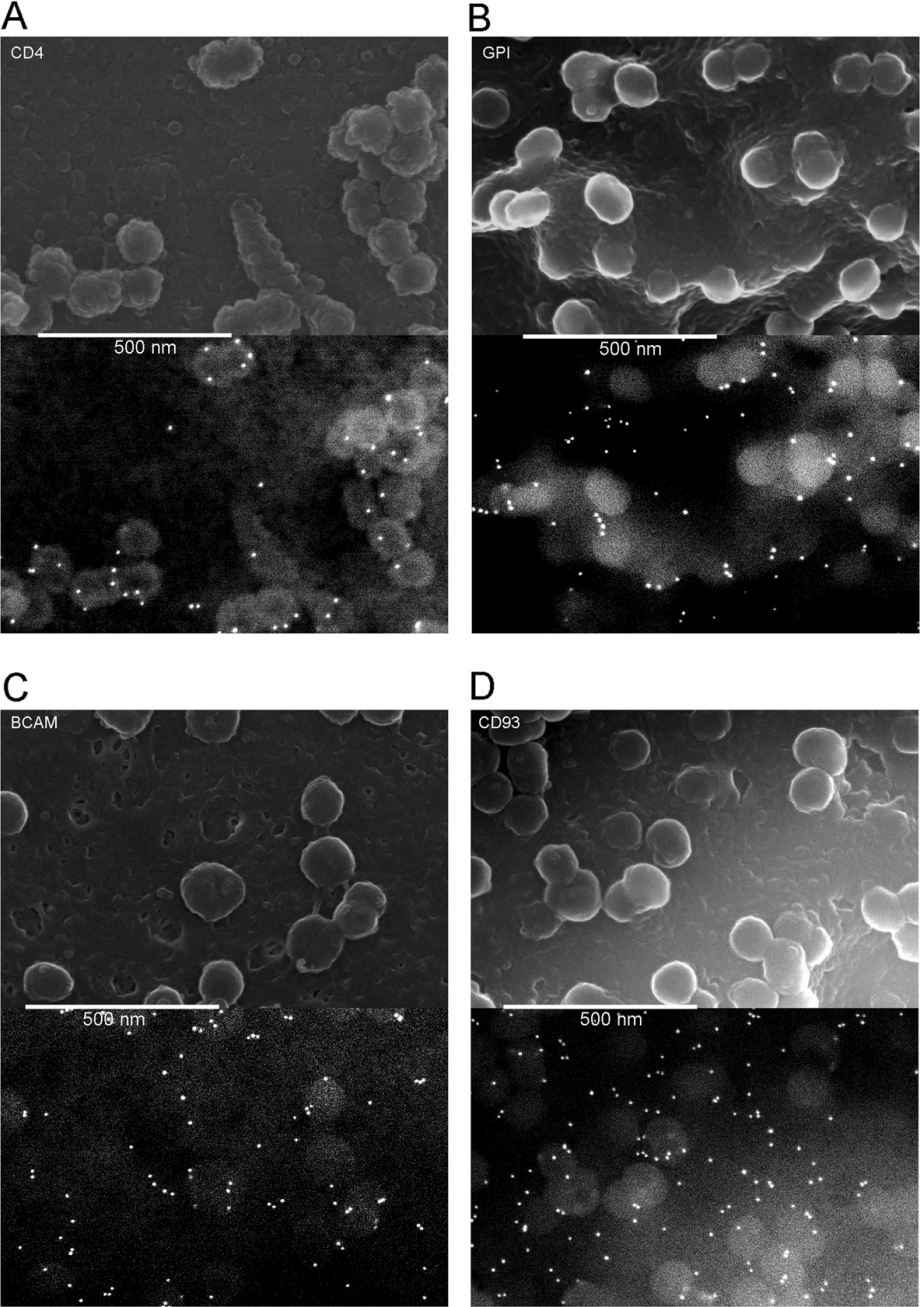
**Recruitment of cellular proteins to viral particles.** SEM was performed as before with 10 nm immunogold labeling of the GFP tag of **A)** CD4, **B)** GPI, **C)** BCAM or **D)** CD93. Some of the immunogold particles are out of the plain of focus, thus leading to different apparent size and intensity.

**Figure 7:**
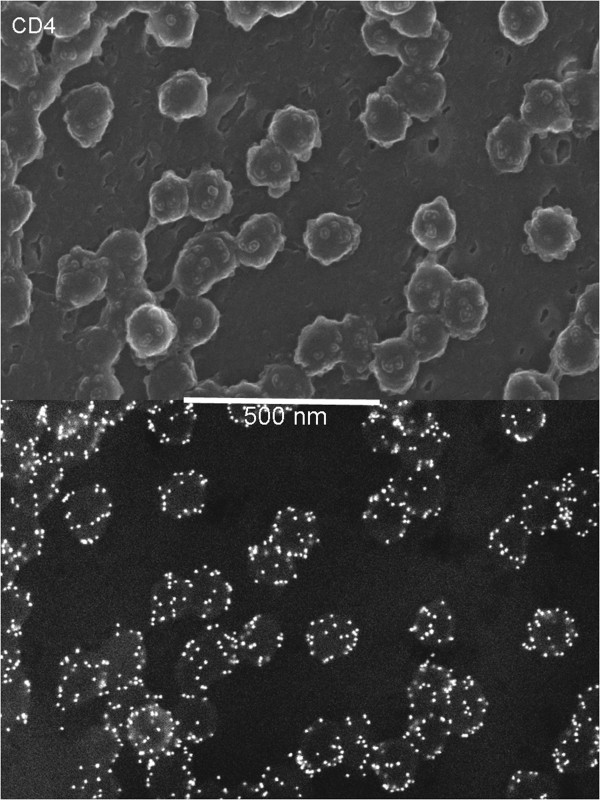
**Wildtype CD4 recruitment to viral particles.** SEM was performed as before using wildtype CD4 and a late domain mutant of HIV-1 Gag. An anti-CD4 antibody conjugated to biotin and a 12 nm gold anti-biotin antibody were used to label CD4.

Given that we observed efficient co-packaging only amongst actively recruited viral glycoproteins, and that CD4 was efficiently co-packaged with VSV-G, we expected CD4 and perhaps BCAM to be actively recruited to viral assembly sites and the other cellular proteins to be inefficiently recruited or perhaps even excluded. To see if these predictions were true, we performed SEM as before with the GPF-tagged CD4, GPI anchor, BCAM and CD93 using an anti-GFP primary antibody (Figure 6A-D). CD4 was indeed seen enriched on budding virions while the distributions of the other glycoproteins were more random. Thus the pattern observed with the viral glycoproteins was reinforced with the cellular proteins. Because CD4 plays a critical role in the HIV-1 life cycle, we decided to see if untagged wildtype CD4 was similarly recruited (Figure 7). For this imaging we used a late domain mutant Gag and a biotin-conjugated anti-CD4 primary antibody. Under these conditions the CD4 was labeled very efficiently and was strikingly localized to viral particles, indicating that CD4 is efficiently and actively incorporated into virus during HIV-1 budding.

## Conclusions

Our data demonstrate that various glycoproteins can be efficiently co-packaged into the same individual virion only when the glycoproteins are actively recruited to assembly and budding sites, while glycoproteins that are not actively recruited are not efficiently co-packaged. The compatibility of recruitment of different glycoproteins is consistent with a mechanism of recruitment that is the same for diverse viruses, but does not rule out dissimilar compatible mechanisms. Whereas co-packaging incompatibility indicates different mechanisms of glycoprotein recruitment. Interestingly, cellular proteins can also be actively recruited, though of the cellular proteins that we assayed, only CD4 was actively recruited. Further testing would be needed to determine if active recruitment of cellular proteins is as rare as our data suggest. Regardless, the identification of classes of proteins that are and are not actively recruited may aid in identifying common features that facilitate active recruitment and aid in elucidating the mechanisms by which they function.

## Methods

### Cells, plasmids and antibodies

HEK-293 FT (Invitrogen), 293 T mCAT-1 (Walter Mothes, Yale University), 293 T TVA (John Young, Scripps Research Institute) and HeLa TZM-bl (NIH AIDS Reagent Program, Division of AIDS, NIAID, NIH: Dr. John C. Kappes, Dr. Xiaoyun Wu and Tranzyme Inc.) were maintained in DMEM supplemented with 10% fetal bovine serum, 2 mM glutamine and 10 mM vitamins. For HIV infectivity we used a pNL4-3 derivative without *Env*, *Vpu*, *Vpr*, *Vif* and *Nef*, and with a reverse intron interrupted *Gaussia* luciferase reporter, HIV-Gluc [32]. For SEM we used a similar pNL4-3 derivative that has Puro-Cherry as the reporter and with mutations in both the late domain and PR active site or a CMV driven late domain mutant Gag previously described [4]. For viral glycoproteins we used plasmids expressing codon optimized consensus B-clade HIV Env (Beatrice Hahn, University of Pennsylvania [45]), MLV Env with a YFP tag in the SU subunit (Walter Mothes, Yale University [46]), RSV Env (Eric Hunter, Emory University [47]), VSV-G (NIH AIDS Reagent Program, Division of AIDS, NIAID, NIH: Dr. Lung-Ji Chang [48]) and flag tagged Ebola GP with the mucin domain deleted (tag added to plasmid from David Sanders, Perdue University [49]). Entry defective versions of VSV-G and Ebola GP were made by introducing A117R mutation or G87A and F88A mutations into VSV-G or Ebola GP, respectively, by standard cloning techniques [34,35]. For the cellular glycoproteins we cloned the cDNA coding sequence for post-leader peptide sequence of CD4 (Nathaniel Landau, New York University), BCAM (GenBank: BC050450.1), CD93 (GenBank: BC028075.1), GPC3 (GenBank: BC035972.1), NPR (GenBank: BC131540.1) or the GPI anchor peptide from CD55 (GenBank: NM_000574.3) downstream of GFP with an influenza HA leader peptide using standard cloning procedures. A CMV driven wildtype CD4 IRES GFP plasmid was used for the SEM in Figure 7. For capture of virions we used 2G12 against HIV Env (NIH AIDS Reagent Program, Division of AIDS, NIAID, NIH: Dr. Hermann Katinger), polyclonal rabbit anti-GFP for MLV Env and cellular glycoproteins, 8C5.4 against RSV Env (Eric Hunter, Emory University [50]), 8G5F11 against VSV-G (hybridoma, KeraFAST) and M2 anti-flag (F1804 Sigma) for Ebola GP. Surface labeling of GFP was done using Alexa Fluor 647 conjugated rabbit anti-GFP (Invitrogen). For SEM monoclonal anti-GFP 20 mouse antibody (G6539 Sigma), 2G12 anti-HIV-1 Env antibody and anti-CD4 biotin conjugate antibody (MHCD0415 Caltag) were used for primary labeling. For secondary labeling, 10 nm or 18 nm gold conjugated anti-mouse, 10 nm anti-human and 12 nm anti-biotin antibodies were used (EM.GMHL10 BBI, 115-215-146 Jackson Laboratories, EM.GAHL10 BBI, EM.GAB12 BBI).

### Co-capture assay

To produce virus to use in the capture assay HEK-293 FT cells were transfected with HIV-Gluc (500 ng) and the viral glycoprotein plasmids so that the viral glycoproteins would be in a limiting amount (10 ng, 5 ng, 20 ng, 10 ng and 10 ng for HIV, MLV, RSV, VSV and Ebola, respectively) using polyethylenimine. These amounts were arrived at empirically from titration curves of each glycoprotein in order for the infectivity to be similar for all of them, though variability existed between subsequent experiments (data not shown). For the cellular glycoproteins the amounts used are indicated in Figure 5 and represent amounts that had similar levels of surface labeling as 5 ng of the MLV Env plasmid. Two days after transfection the supernatants were collected and processed with one freeze-thaw cycle. Antibodies were bound to the wells of an ELISA plate by adding 20 μL of PBS with or without antibody to the wells and incubated overnight at 4°C. The overlay was then aspirated and 100 μL of blocking buffer (PBS with 1% BSA, 5% sucrose & .05% sodium azide) was incubated in the wells for one hour. The blocking buffer was then removed and 10 μL of supernatant with equal volume of PBS was added to the wells. Following a three hour incubation with virus at 37°C, the supernatant was removed and all wells except those meant for positive infectivity controls were washed twice with 100 μL of PBS. 20 μL of PBS was then added to the washed wells to normalize the volume to the positive controls and then the relevant cells were plated into the wells for infection. Two days after infection, 20 μL of supernatant from each well was transferred to a black 96 well plate and assayed for luciferase activity by adding 25 μL of coelenterazine (NanoLight Technology) and reading the luminescence with a Turner Biosystems’ Veritas luminometer. Statistical p values were calculated by performing a one-sample t-test on the natural logarithm of (antibody capture signal/no antibody capture signal) from each experiment.

### Cellular GFP detection

Cells were transfected as for the capture assay and two days later, after the removal of the supernatant, the cells were washed with PBS and then incubated with 10 mM EDTA in PBS. Once the cells had disassociated they were transferred to tubes with 5% rabbit serum for blocking. Samples were kept at 4°C for all subsequent steps until fixation. Following 30 minutes of blocking, the cells were pelleted by centrifugation and then incubated with the Alexa Fluor 647 anti-GFP antibody for one hour in 10 mM EDTA PBS. Cells were then gently pelleted again and washed with PBS by resuspending and pelleting, and then fixed in 4% paraformaldehyde in PBS for at least 20 minutes. The fixed cells were then pelleted and resuspended in PBS before being analyzed on an Accuri C6 flow cytometer to detect total GFP fluorescence and surface GFP labeled with the Alexa Fluor antibody.

### Scanning electron microscopy

Imaging of cells by SEM was done as previously described [4]. Briefly, 293 T mCAT-1 or 293 T TVA cells were plated onto glass coverslips with a thin-layer gold coat in a grid pattern and transfected with the late domain mutant and glycoprotein plasmids using polyethylenimine. Fluorescence microscopy was used to map transfected cells prior to fixation and antibody labeling of the glycoproteins. After critical point drying and carbon evaporation coating, samples were imaged with a Hitachi S4700 FE SEM at the University of Missouri Electron Microscopy Core Facility. Image brightness and contrast were adjusted in Microsoft PowerPoint for clear resolution of topology and gold particles.

## Abbreviations

HIV-1: Human immunodeficiency virus; RSV: Rous sarcoma virus; MLV: Murine leukemia virus; VSV-G: Vesicular stomatitis virus glycoprotein; CTD: Cytoplasmic tail domain; MA: Matrix; HEK: Human embryonic kidney; TIP: Tail-interacting protein; SEM: Scanning electron microscopy; ELISA: Enzyme-linked immunosorbent assay; mCAT-1: Mouse cationic amino acid transporter-1; Ebola GP: Ebola glycoprotein; BCAM: Basal cell adhesion molecule; GPC3: Glypican 3; NPR: Natriuretic Peptide Receptor C.

## Competing interests

The authors declare that they have no competing interests.

## Authors’ contributions

DAG participated in the design and execution of SEM, co-capture and surface labeling experiments, and drafted the manuscript. GYO and TML participated in design and execution of SEM, and contributed to revising the manuscript. MCJ conceived of the study, participated in its design and contributed to revising the manuscript. All authors read and approved the final manuscript.
